# Regioselective Glucuronidation of Diosmetin and Chrysoeriol by the Interplay of Glucuronidation and Transport in UGT1A9-Overexpressing HeLa Cells

**DOI:** 10.1371/journal.pone.0166239

**Published:** 2016-11-10

**Authors:** Xuejun Zeng, Jian Shi, Min Zhao, Qingwei Chen, Liping Wang, Huangyu Jiang, Feifei Luo, Lijun Zhu, Linlin Lu, Xinchun Wang, Zhongqiu Liu

**Affiliations:** 1 Department of Pharmacy, First Hospital Affiliated to Shihezi University, Shihezi, Xinjiang, 832002, China; 2 International Institute for Translational Chinese Medicine, Guangzhou University of Chinese Medicine, Guangzhou, Guangdong, 510006, China; University of Insubria, ITALY

## Abstract

This study aimed to determine the reaction kinetics of the regioselective glucuronidation of diosmetin and chrysoeriol, two important methylated metabolites of luteolin, by human liver microsomes (HLMs) and uridine-5′-diphosphate glucuronosyltransferase (UGTs) enzymes. This study also investigated the effects of breast cancer resistance protein (BCRP) on the efflux of diosmetin and chrysoeriol glucuronides in HeLa cells overexpressing UGT1A9 (HeLa—UGT1A9). After incubation with HLMs in the presence of UDP-glucuronic acid, diosmetin and chrysoeriol gained two glucuronides each, and the OH—in each B ring of diosmetin and chrysoeriol was the preferable site for glucuronidation. Screening assays with 12 human expressed UGT enzymes and chemical-inhibition assays demonstrated that glucuronide formation was almost exclusively catalyzed by UGT1A1, UGT1A6, and UGT1A9. Importantly, in HeLa—UGT1A9, Ko143 significantly inhibited the efflux of diosmetin and chrysoeriol glucuronides and increased their intracellular levels in a dose-dependent manner. This observation suggested that BCRP-mediated excretion was the predominant pathway for diosmetin and chrysoeriol disposition. In conclusion, UGT1A1, UGT1A6, and UGT1A9 were the chief contributors to the regioselective glucuronidation of diosmetin and chrysoeriol in the liver. Moreover, cellular glucuronidation was significantly altered by inhibiting BCRP, revealing a notable interplay between glucuronidation and efflux transport. Diosmetin and chrysoeriol possibly have different effects on anti-cancer due to the difference of UGT isoforms in different cancer cells.

## Introduction

Luteolin (3′,4′,5,7-tetrahydroxyflavone), a typical catecholic flavonoid, is present in various dietary sources such as fruits, vegetables, wines, oils, tea etc [1]. It exhibits a wide range of pharmacological effects, including antioxidation, anti-inflammation, and anticarcinogenic activity [2,3]. A study reported that after luteolin’s oral administration, it undergoes methylation as a major metabolic pathway. Isomers diosmetin (5,7,3′-trihydroxy-4′-methoxyflavone) and chrysoeriol (5,7,4′-trihydroxy-3′- methoxyflavone) are the two methylated metabolites [4] of luteolin. These isomers have been shown to exhibit common biological effects, such as osteoporosis prevention [5]. Diosmetin is an active ingredient of some medications [6] and has been reported to display several biological properties, including anticancer effects [7,8] and antibacterial actions [9]^-^. Similar to diosmetin, chrysoeriol is mainly distributed in many plant products, such as parsley [10] and peanut hull [11]. Chrysoeriol could inhibit lipid peroxidation in low-density lipoprotein [11] and exhibit antioxidant activity and free radical scavenging ability [12]. The biological properties of flavone are well known to be severely limited by the compound’s low bioavailability resulting from extensive metabolism and excretion. However, studies on the mechanism and characteristics of the metabolism and excretion of diosmetin and chrysoeriol are few.

Uridine-5′-diphosphate glucuronosyltransferases (UGTs) are a superfamily of enzymes that catalyze the glucuronidation of many compounds [13]. Glucuronidation, a primary phase II conjugation reaction, is considered as a detoxification mechanism because the generated glucuronides are highly polar and can be rapidly eliminated [14,15]. UGT isoforms involved in glucuronidation of phenolics and other relevant compounds discussed belong to UGT1A or UGT2B family [13], and these enzymes possess broad and overlapping substrate specificities. Our previous study demonstrated that diosmetin and chrysoeriol could be metabolized to their phase II metabolites by Ugts [16]. Thus, the UGT-catalyzed glucuronidation could play a key role in determining the bioavailability and clearance of diosmetin and chrysoeriol [13]. However, the characteristics of and difference between the glucuronidation of diosmetin and chrysoeriol by UGTs and the other major contributing enzymes have not been fully established. This knowledge is valuable in achieving better prediction of diosmetin and chrysoeriol disposition, which could be the main factors affecting the compounds’ bioavailability and biological activities. Our findings could also add to the general understanding of the mechanisms of action of diosmetin and chrysoeriol *in vivo*.

On the other hand, the high polarity of the glucuronides renders difficult their passive diffusion outward from the cell. As such, the glucuronides require efflux transporters to exit the cells. The cellular glucuronidation of flavone is hence presumed to be affected by the activities of glucuronidation enzymes, relevant efflux transporters, and the interplay between UGT enzymes and efflux transporters [17]. Breast cancer resistance protein (BCRP), an important efflux pump in the cell membrane, is known to participate in the biliary and intestinal excretion of many flavone glucuronides [18–20]. Therefore, the interplay between BCRP and UGT enzymes could play a key role in the phase II disposition of flavone. Understanding the role of BCRP is important for assessing the contribution of BCRP in glucuronide disposition. Our group studied the effect of such interplay on luteolin disposition using established HeLa—UGT1A9 cells with UGT1A9 and BCRP to investigate the metabolism of luteolin and the excretion of its glucuronides [13]. Compared with Caco-2 and MDCK II cells, HeLa—UGT1A9 cells serve as a suitable model for the study of the interplay between transporters and UGT enzymes because of the functional expression of only one dominant UGT and one dominant efflux transporter in these cells [21,22]. Diosmetin and chrysoeriol are the main methylation metabolites of luteolin. Hence, the study of the effect of the interplay between transporters and UGT enzymes on diosmetin and chrysoeriol metabolism is important and useful for determining the mechanism and characteristics of the absorption and metabolism of luteolin *in vivo*.

The present study aimed to determine and compare the reaction kinetics of the regioselective glucuronidation of diosmetin and chrysoeriol by human liver microsomes (HLMs) and expressed UGT enzymes. The main UGT enzymes involved in such regioselective glucuronidation were identified and determined through incubation with a panel of 12 UGT enzymes and HLMs in the presence of specific UGT inhibitors. Kinetic parameters were derived by fitting and appropriate modeling of the data. UGT1A9 was one of important UGT enzymes in the liver involved in the metabolism of diosmetin and chrysoeriol as determined in this study. Hence, we used HeLa—UGT1A9 cells to explore how the interplay between UGT1A9 and BCRP affects diosmetin and chrysoeriol disposition. The results of our study would contribute to improving the understanding on the absorption and disposition of luteolin.

## Material and Methods

### Chemicals and Reagents

Diosmetin (>98% purity) was purchased from Chengdu Must Pharmaceutical Co. Ltd. (Chengdu, China). Chrysoeriol was acquired from Shenzhen Zhenqiang Bio-Technology Co. Ltd. (Shenzhen, China). Tilianin (95% purity; internal standard; IS) was provided by the Department of Pharmacy, First Hospital Affiliated to Shihezi University. Diosmetin-7-O-glucuronide (Dio-7-G), diosmetin-3′-O-glucuronide (Dio-3′-G), chrysoeriol-7-O-glucuronide (Chr-7-G) and chrysoeriol-4′-O-glucuronide (Chr-4′-G) were prepared and identified by our laboratory [16]. Carvacrol and troglitazone were purchased from Qiyun Bio-Technology Co. Ltd. (Guangzhou, China). Phenylbutazone, bilirubin, UDP glucuronic acid (UDPGA), alamethicin, MgCl_2_, D-saccharic-1,4-lactone monohydrate, β-glucuronidase (Type HP-2 from *Helix pomatia*), Hanks’ Balanced Salts, HEPES (N-(2-Hydroxyethyl) piperazine-N'-(2-ethanesulfonic acid)), MTT and Ko143 were purchased from Sigma—Aldrich Co. (St. Louis, MO, USA). Human expressed UGTs (Supersomes Enzymes), pooled HLMs (35 individuals), and human expressed UGT enzymes (UGT1A1, 1A3, 1A4, 1A6, 1A7, 1A8, 1A9, 1A10, 2B4, 2B7, 2B15, and 2B17) were purchased from BD Biosciences (Woburn, MA, USA). HeLa cells and HeLa cells stably transfected with UGT1A9 (HeLa—UGT1A9 cells) were provided by Dr. Ming Hu (Department of Pharmacological and Pharmaceutical Sciences, College of Pharmacy, University of Houston, Houston, Texas, USA). HyClone fetal bovine serum and HyClone penicillin—streptomycin solution were obtained from Thermo Fisher Scientific (MA, USA). All other chemicals and solvents were of analytical grade or better.

### Cell Culture

HeLa and HeLa—UGT1A9 cells were seeded into six-well plate at a density of 1.0 × 10^5^ cells/well, and were grown using Dulbecco’s modified Eagle’smedium (DMEM) supplemented with 10% fetal bovine serum (FBS). After 3 ~ 4 days of seeding, the cells can be used for the glucuronidation experiments.

### Glucuronidation Assay

All metabolism studies in vitro were approved by the First Hospital Affiliated to Shihezi University Research Ethics Committee. The glucuronidation assay was performed as described in previous publications [23–25]. The stock solutions of diosmetin or chrysoeriol were prepared in methanol-dimethyl sulfoxide (DMSO) (1:1, v/v) and diluted with methanol—DMSO (1:1, v/v) to the desired concentrations immediately before use. The incubation volumes were 120 μL, and the total organic solvent content was 1%. The mixtures contained HLMs or expressed UGT enzymes, MgCl_2_ (0.88 mM), saccharolactone (4.4 mM), alamethicin (22 μg/mL), and diosmetin or chrysoeriol (at desired concentrations) in 50 mM potassium phosphate (pH 7.4). The reactions were initiated by adding UDPGA (3.5 mM) at 37°C and then allowed to stand for 30 min in a water bath. These reactions were terminated by adding 60 μL ice-cold acetonitrile containing the IS (25 nM tilianin). The samples were centrifuged at 13,000 rpm for 30 min, and the supernatant was analyzed by ultra high pressure liquid chromatography (UHPLC)–tandem mass spectrometry (MS/MS). All experiments were performed in triplicate.

### Kinetic Evaluation

The enzyme kinetic parameters were obtained by fitting kinetic models to the experimental data using GraphPad Prism version 5.04 for Windows (GraphPad Software Inc., San Diego, CA, USA). The best model was selected based on visual inspection of the Eadie-Hofstee plots, the calculated *r*^2^ values, and corrected Akaike’s information criterion [26]. The HLMs or human expressed UGT enzymes were employed for kinetic studies at concentrations ranging from 0.0021 mg/mL to 0.0053 mg/mL. The rates of the glucuronidation were fitted to the following equations:

$$V\;=\;\frac{C_{\text{metabolite}}}{t\times C_{\text{protein}}}$$

Where *V* (nmol/mg/min) is the rates of the glucuronidation, *C*_metabolite_ (μM) and *C*_protein_ (mg/mL) respectively are the concentration of the metabolite and the reaction protein, *t* (min) is the reaction time [2,22].

Model selection was based on visual inspection of Eadie—Hofstee plots [26] and it was reported in our previous study [25]. In brief, if the Eadie—Hofstee plot was linear, formation rates (*V*) of glucuronides at different substrate concentrations (C) were fitted to the standard Eq (1):

$$V\;=\;\frac{V_{\text{max}}\times \text{C}}{{\text{K}}_{\text{m}}+\text{C}}$$ (1)

where K_m_ is the Michaelis-Menten constant and *V*_max_ is the maximum rate of glucuronidation. The intrinsic clearance (CL_int_) was derived by *V*_max_/K_m_.

When Eadie—Hofstee plots revealed substrate inhibition kinetics, the reaction rate (*V*) were fitted to Eq (2):

$$V\;=\;\frac{V_{\text{max}1}}{1+\left({\text{K}}_{\text{m}1}/\text{C}\right)+\left(\text{C}/{\text{K}}_{\text{si}}\right)}$$ (2)

where C is the substrate concentration, *V* is the initial reaction rate, *V*_max1_ is the maximum enzyme velocity, K_m1_ is the substrate concentration required to achieve 50% of *V*_max1_, and K_si_ is the substrate inhibition constant. The intrinsic clearance (CL_int_) was derived by *V*_max1_/K_m1_.

### Assay with Human Expressed UGTs

The method of this study as described in detail previously [25]. In this study, 12 human expressed UGTs (UGT1A1, 1A3, 1A4, 1A6, 1A7, 1A8, 1A9, 1A10, 2B4, 2B7, 2B15, and 2B17) were used for the glucuronidation of diosmetin and chrysoeriol. Three substrate concentrations (1.25, 2.5, and 10 μM) were employed in this experiment, and the incubation progress was performed as shown above. The incubation conditions were as follows: protein concentration: 0.0053 mg/mL; temperature of water: 37°C; shaker speed: 50 rpm; reaction time: 30 min. After incubation, all samples were pretreated as described above and analyzed by UHPLC—MS/MS.

### Chemical Inhibition Studies

Bilirubin (UGT1A1 inhibitor)[12,27], phenylbutazone (UGT1A6 inhibitor)[25,28], or carvacrol (UGT1A9 inhibitor)[17,29] were used to determine glucuronidation activities of diosmetin and chrysoeriol in the HLMs and expressed UGTs (UGT1A1, UGT1A6, and UGT1A9). The protein concentrations of the HLMs and expressed UGTs were 0.0021–0.0106 mg protein/mL. All of the incubations were run at 37°C for 30 min, and the way of pretreated and determined samples were as discribed above.

### Glucuronides Excretion Experiments in the HeLa and HeLa—UGT1A9 Cell Model

Glucuronides excretion experiments were performed as described in detail previously [17]. The HeLa cells were used to incubate diosmetin and chrysoeriol as the control. Brifely speaking, the cells were incubated at 37°C with loading solution (2 mL). The loading solution were HBSS (9.801 g Hanks’ balanced salts, 0.372 g NaHCO_3_, 3.502 g glucose, 5.963 g HEPES and 1.164 g NaCl dissolved in 900 mL deionized water) which containing different diosmetin or chrysoeriol concentrations (1.25, 2.5, 5, 10, 20, and 40 μM with 0.1% DMSO) at the pre-set time, 500 μL of incubation medium from each well was collected, and the same volume of diosmetin or chrysoeriol solution was loaded in each well. Ko143, whenever applied, a chemical inhibitor of BCRP (K_i_ < 1 μM [30]), was used to determine whether the diosmetin or chrysoeriol glucuronides were the substrates of BCRP. Ko143 (5 or 10 μM) was diluted in a solution containing diosmetin or chrysoeriol (2.5 μM). The collected samples were mixed with 250 μL acetonitrile containing 25 nM tilianin and then analyzed by UHPLC—MS/MS after centrifugation at 13,000 rpm for 30 min. After the excretion experiment, the cells were washed and selected in 300 μL HBSS buffer, and then the cells were ultrasonicated. After centrifugation at 13,000 rpm for 30 min, the 200 μL supernatant was obtained and mixed with 100 μL acetonitrile containing 25 nM tilianin. The mixture was then centrifuged at 13,000 rpm for 30 min and prepared for subsequent UHPLC—MS/MS analysis.

The excretion rates, glucuronides intracellular, Glucuronides excretion, the clearance of efflux transporter (CL) and the fraction of the metabolized dose (*f*_met_) experiments were calculated as described in detail previously [17].

### MTT assay

The cytotoxicity of diosmetin and chrysoeriol on A549 and HepG2 cells was measured using the MTT assay. Cells were seeded at a density of 3 × 10^3^/well in 96-well plates and grown overnight, and then the medium was replaced with a fresh medium containing substrates (5 μM Ko143, 10 μM Ko143, 10 μM diosmetin or chrysoeriol contained or not contained 5 μM Ko143 or 10 μM Ko143). The cells were further incubated at 37°C with 5% CO_2_ for 48 h. The culture medium was removed and replaced with 100 μL 0.5 mg/mL 3-(4, 5-dimethylthiozol-2-yl)-3, 5-dipheryl tetrazolium bromide (MTT) solution and incubated at 37°C for 4 h. The supernatants were discarded, and 150 μL of dimethyl sulfoxide was added to each well. The plates were shaken for 10 min at room temperature, and then, the stained formazan product was determined at 570 nm with a multimode plate reader.

### UHPLC—MS/MS system

Quantification of the analytes was carried out on an Agilent 1290 UHPLC system coupled to Agilent 6490 triple quadrupole MS/MS. The conditions were as follows: column, Acquity UPLC HSS T3, 1.8 μm, 2.1 mm × 100 mm (Waters, USA); mobile phase A, 100% aqueous buffer (0.1%, v/v formic acid, pH 2.5); mobile phase B, 100% acetonitrile; flow rate, 0.3 mL/min; gradient for diosmetin and its metabolites, 0–1 min, 25–25% B, 1–3.5 min, 25–33% B, 3.5–5 min, 33–90% B, 5–6 min, 90–90% B, 6–7 min, 90–21% B; gradient for chrysoeriol and its metabolites, 0–5 min, 21–21% B, 5–7 min, 21–90% B, 7–8 min, 90–90% B, 8–9 min, 90–21% B; and injection volume, 2 μL.

The mass spectrometric detection in this study was performed using an ESI source in positive ionization modes, with capillary voltage set at 3.0 kV, sheath gas and desolvation temperatures set at 300 and 250°C, respectively. The sheath gas and desolvation flows were 11 and 14 L/min, respectively. The nozzle voltage was set at 1.5 kV. Acquisition was performed in a multiple-reaction monitoring (MRM) mode at m/z transitions of 447.41→285.20 for tilianin; 477.00→301.00 for Dio-7-G, Dio-3′-G, Chr-7-G, and Chr-4′-G; and 301.27→286.10 for diosmetin and chrysoeriol in the positive ESI mode. Collision energies were 20 V for tilianin, 24 V for diosmetin or chrysoeriol, and 32 V for diosmetin glucuronides or chrysoeriol glucuronides, respectively.

### Statistical Analysis

One-way ANOVA with or without Tukey—Kramer multiple comparison (post hoc) tests were used to evaluate statistical difference. Data were expressed as mean ± standard deviation (S.D) (n = 3), and the level of significance was set at *p* < 0.05 (“*” or “^#^”), *p* < 0.01 (“**” or “^##^”), or *p* < 0.001 (“***” or “^###^”).

## Results

### Analysis of Diosmetin and Chrysoeriol and their Metabolites by UHPLC—MS/MS

**Fig 1:**
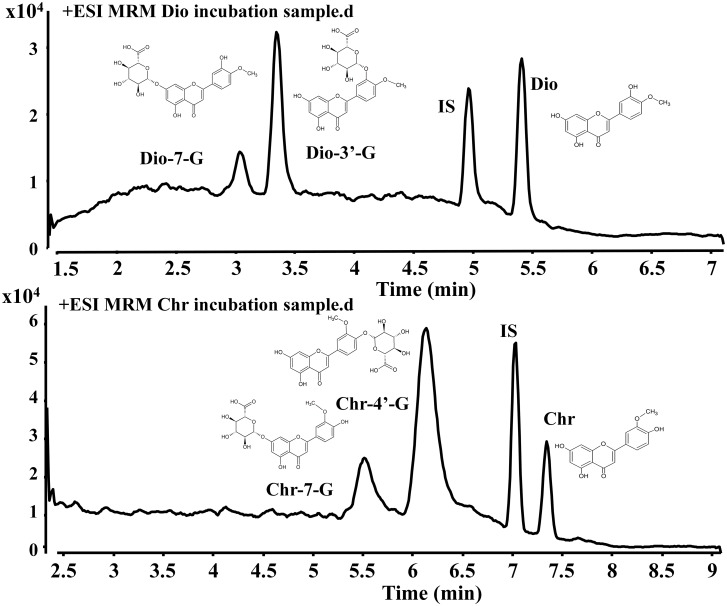
Analysis of diosmetin, chrysoeriol, and their metabolites by UHPLC—MS/MS. Fig 1a and 1b show the chromatogram of the incubation samples of diosmetin and chrysoeriol by HLMs, respectively.

We already identified the metabolites of diosmetin and chrysoeriol in our previous study [16]. UHPLC—MS/MS analysis showed that two mono-glucuronides were formed in HLMs incubations with diosmetin and chrysoeriol in the presence of UDPGA. The retention times of diosmetin and its metabolites (Dio-7-G and Dio-3′-G) were 5.47, 3.06, and 3.38 min, respectively (Fig 1a). The peak eluting times at 7.35, 5.54, and 6.21 min corresponded to those of chrysoeriol and its metabolites (Chr-7-G and Chr-4′-G) (Fig 1b).

### Kinetics of Diosmetin and Chrysoeriol Glucuronidation by HLMs

**Fig 2:**
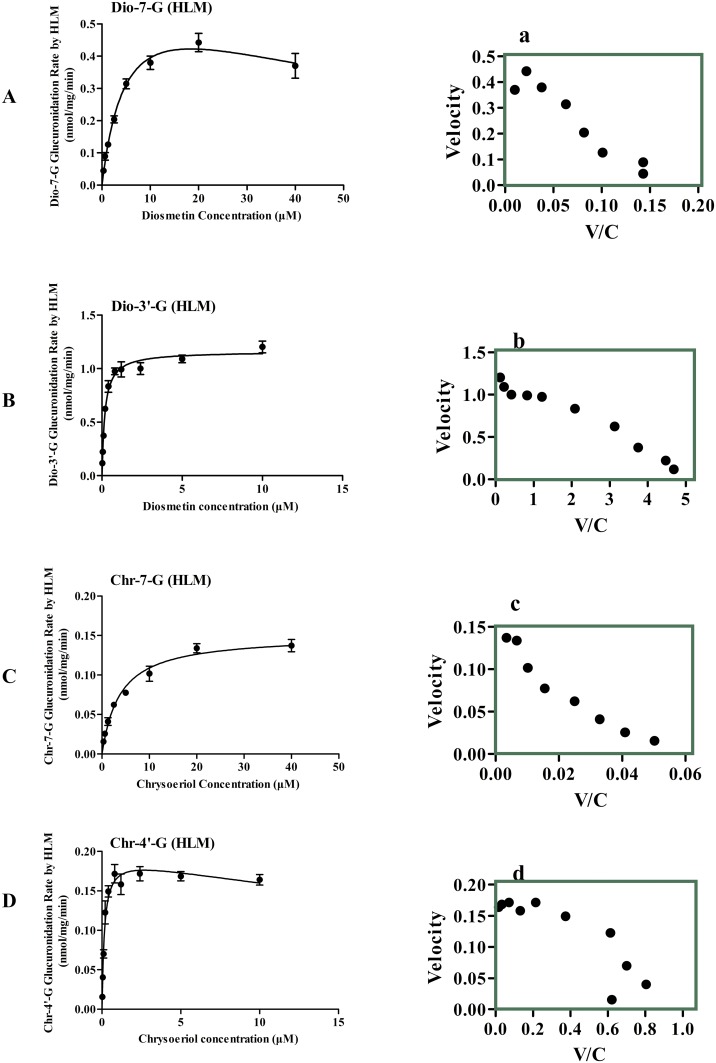
Kinetics of diosmetin and chrysoeriol glucuronidation by HLMs. The curves were estimated on the basis of fitted parameters generated using the Michaelis-Menten kinetics (B, C) or substrate inhibition (A, D) in HLMs. The Eadie—Hofstee plots are shown in right panal (a-d). Each data point corresponds to the average of three determinations with error bars representing S.D.

Diosmetin and chrysoeriol can be metabolized by HLMs into two metabolites, respectively. Dio-7-G and Dio-3’-G were the metabolites of diosmetin, and the rate of formation Dio-3′-G was much faster than that of Dio-7-G in HLMs within the tested concentration ranges. Hence, the preferred site for the catalysis of diosmetin glucuronidation was 3′-OH rather than 7-OH. The Eadie—Hofstee plots (Fig 2a and 2b) were used as evidences to state that the glucuronidation of 7-OH glucuronidation exhibited substrate inhibition profiles (Fig 2A), whereas the glucuronidation of 3′-OH followed the classic Michaelis—Menten kinetics (Fig 2B). Chr-7-G and Chr-4′-G were the metabolites of chrysoeriol, and the rate of formation of Chr-4′-G was much faster than that of Chr-7-G in the HLMs within the tested concentration ranges. Therefore, the preferred site for the catalysis of chrysoeriol glucuronidation was 4′-OH rather than 7-OH. The Eadie—Hofstee plots (Fig 2c and 2d) were used as evidences to state that the glucuronidation of 7-OH glucuronidation exhibited the classic Michaelis—Menten kinetics (Fig 2C), whereas 4′-OH followed substrate inhibition profiles (Fig 2D).

The kinetic parameters of the glucuronidation of diosmetin and chrysoeriol by HLMs are shown in Table 1. Dio-3′-G in the HLMs exhibited a much lower K_m_ value (0.19 μM for Dio-3′-G, 4.66 μM for Dio-7-G). The CL_int_ of diosmetin in the HLMs through 3′-OH was about 48 times that of the 7-OH pathway (6.25 mL/min/mg for Dio-3′-G, 0.13 mL/min/mg for Dio-7-G). On the other hand, the HLMs exhibited a much lower K_m_ of Chr-4′-G than of Chr-7-G (0.15 μM for Chr-4′-G, 3.83 μM for Chr-7-G). As such, the CL_int_ of chrysoeriol in the HLMs through 4′-OH was about 32 times that of the 7-OH pathway (1.27 mL/min/mg for Chr-4′-G, 0.04 mL/min/mg for Chr-7-G).

**Table 1:** Kinetic parameters of diosmetin and chrysoeriol glucuronidation by HLMs.

		K_m_ (μM)	*V*_max_	CL_int_
			nmol/mg/min	(mL/min/mg)
diosmetin	Dio-7-G	4.66±0.72	0.61±0.05	0.13
	Dio-3′-G	0.19±0.02	1.16±0.03	6.25
chrysoeriol	Chr-7-G	3.83±0.55	0.15±0.01	0.04
	Chr-4′-G	0.15±0.03	0.20±0.01	1.27

### Main UGTs Responsible for the Glucuronidation of Diosmetin and Chrysoeriol In Vitro

**Fig 3:**
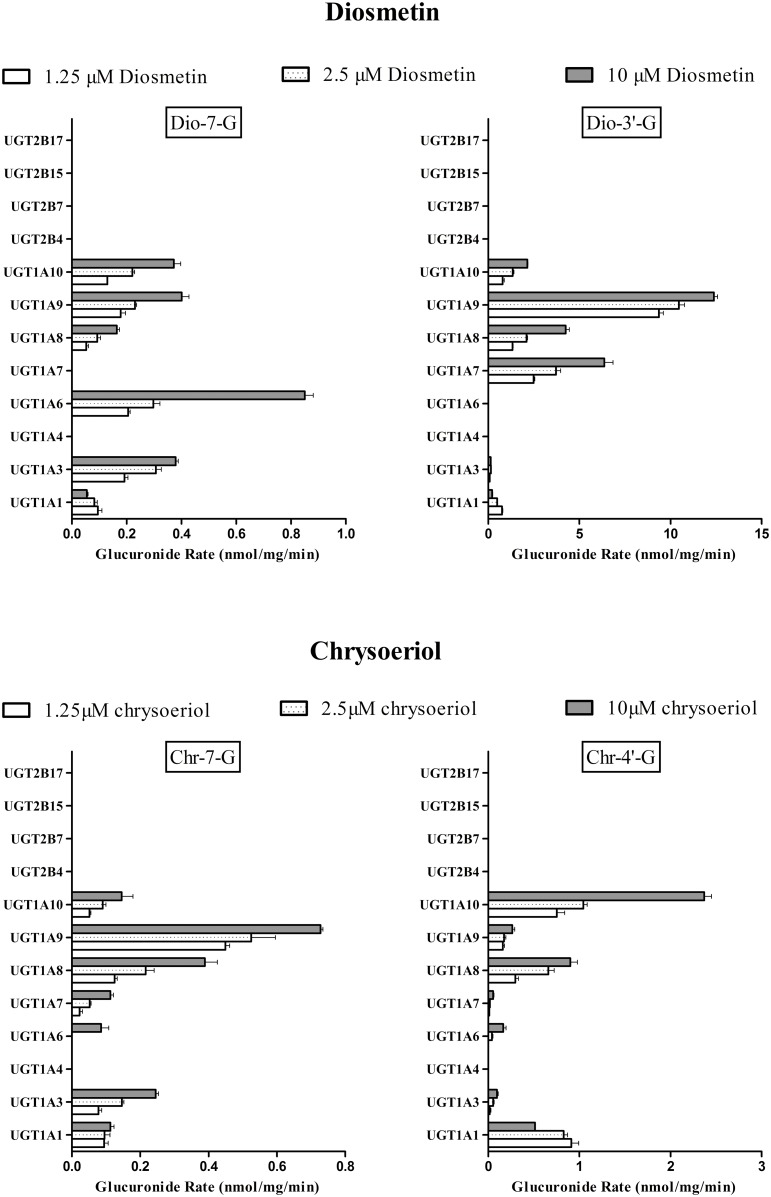
Glucuronidation of diosmetin and chrysoeriol by human expressed UGT isoforms. Experiments were conducted at concentrations 1.25, 2.5, and 10 μM. The concentration of each human expressed UGT isoform was 0.0053 mg/mL. The experiments were conducted at 37°C for 30 min, and the amount of glucuronides formed were measured by UHPLC—MS/MS. The rates of glucuronidation were calculated as nmol/mg/min. Each column corresponds to the average of three determinations with error bars representing the S.D.

We used UGT1A1, UGT1A3, UGT1A4, UGT1A6, UGT1A7, UGT1A8, UGT1A9, UGT1A10, UGT2B4, UGT2B7, UGT2B15, UGT2B17 to explore which UGT isoform was the main isoform in charge of diosmetin or chrysoeriol UGT metabolism (Fig 3). The results showed that the isoforms that produced the most rapid glucuronidation rates of Dio-7-G (0.21 ± 0.007 nmol/mg/min to 0.85 ± 0.03 nmol/mg/min), Dio-3′-G (9.36 ± 0.23 nmol/mg/min to 12.38 ± 0.20 nmol/mg/min), Chr-7-G (0.44 ± 0.01 nmol/mg/min to 0.73 ± 0.01 nmol/mg/min), Chr-4′-G (0.75 ± 0.09 nmol/mg/min to 2.37 ± 0.08 nmol/mg/min) were UGT1A6, UGT1A9, UGT1A9 and UGT1A10, respectively. UGT1A3, UGT1A9, UGT1A10 made a prominent contribution to forming Dio-7-G; UGT1A7 made a prominent contribution to forming Dio-3′-G; UGT1A3 and UGT1A8 made a prominent contribution to the formation of Chr-7-G; UGT1A1 and UGT1A8 made a prominent contribution to the formation of Chr-4′-G. UGT2B family and UGT1A4 had no contribution to diosmetin or chrysoeriol UGT metabolism.

### Effects of Chemical Inhibitor on Diosmetin and Chrysoeriol Metabolism

**Fig 4:**
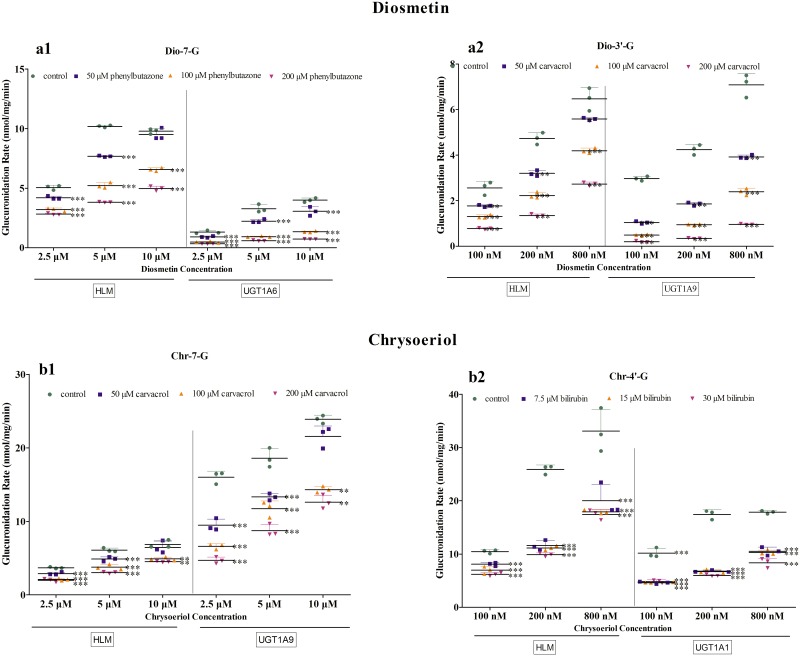
Effects of chemical inhibitors on diosmetin and metabolism and UGT1A9- and UGT1A1-mediated chrysoeriol metabolism in HLMs. Fig 4 A shows the inhibitory effects of phenylbutazone on the 7-O-glucuronide of diosmetin in HLMs and UGT1A6. Fig 4 B displays the inhibitory effects of carvacrol on the 3′-O-glucuronide of diosmetin in HLMs and UGT1A9. Fig 4 C presents the inhibitory effects of carvacrol on the 7-O-glucuronide of chrysoeriol in HLMs and UGT1A9. Fig 4 D shows the inhibitory effects of bilirubin on the 4′-O-glucuronides of chrysoeriol in HLMs and UGT1A1. Each column corresponds to the average of three determinations with error bars representing the S.D. The “*” symbol means a statistically significant difference compared with control at p < 0.05; “**” means p < 0.01; “***” means p < 0.001.

We determined the inhibitory effects of bilirubin (UGT1A1 inhibitor), phenylbutazone (UGT1A6 inhibitor), and carvacrol (UGT1A9 inhibitor) on the glucuronidation of diosmetin (Fig 4a1 and 4a2) and chrysoeriol (Fig 4b1 and 4b2) in the HLMs, UGT1A1, UGT1A6 and UGT1A9. As we can see in Fig 4, the phenylbutazone, calvacrol, calvacrol and bilirubin can significantly decrease the formation of Dio-7-G, Dio-3’-G, Chr-7-G and Chr-4’-G compared with control, respectively. And these chemical inhibitors can dose-dependently inhibited the formation of Dio-7-G, Dio-3’-G, Chr-7-G and Chr-4’-G, which further proved that UGT1A6, UGT1A9, UGT1A9 and UGT1A1 respectively were the main UGTs involved in the formation of Dio-7-G, Dio-3’-G, Chr-7-G and Chr-4’-G in HLMs.

### Kinetics of Diosmetin and Chrysoeriol Glucuronidation by Human Expressed UGT enzymes

**Fig 5:**
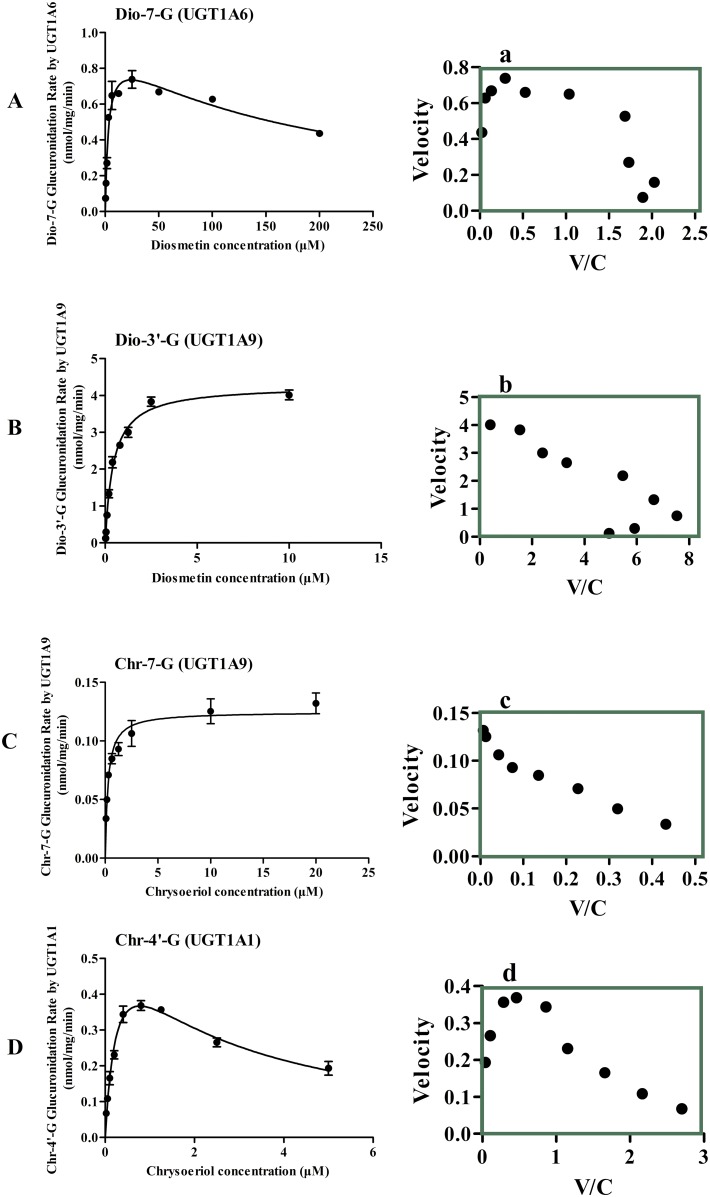
Kinetics of diosmetin and chrysoeriol glucuronidation by human expressed UGT enzymes. The curves are estimated on the basis of fitted parameters generated using the substrate inhibition (A, D) or Michaelis-Menten kinetics (B, C) in UGT1A6, UGT1A1, UGT1A9, and UGT1A9, respectively. The Eadie—Hofstee plots are shown in the right panal (a-d). Each data point corresponds to the average of three determinations with error bars representing the S.D.

According to above results, we used human expressed UGT enzymes to incubate different concentration diosmetin and chrysoeriol and determined their glucuronidation rates. We used UGT1A6 and UGT1A9 to incubate diosmetin to further confirm if they were the main isoform of forming Dio-7-G and Dio-3′-G, respectively; and we used UGT1A9 and UGT1A1 to incubate chrysoeriol to further confirm if they were the main isoform of forming Chr-7-G and Chr-4′-G, respectively. Within the tested concentration ranges, The Eadie—Hofstee plots (Fig 5a and 5d) were used as evidences to state that both UGT1A6- and UGT1A1-mediated the formation of Dio-7-G (Fig 5A) and Chr-4′-G (Fig 5D) exhibited substrate inhibition kinetic characteristics. And the Eadie—Hofstee plots (Fig 5b and 5c) were used as evidences to state that both UGT1A9-mediated formation of Dio-3′-G (Fig 5B) and Chr-7-G (Fig 5C) exhibited classic Michaelis-Menten kinetic characteristics.

The kinetic parameters of the diosmetin and chrysoeriol glucuronidation catalyzed by human expressed UGT enzymes are shown in Table 2. These data accompanied the absence of large variation in K_m_ value of the glucuronides except for Dio-7-G (3.02 μM for Dio-7-G in UGT1A6, 0.45 μM for Dio-3′-G in UGT1A9, 0.26 μM for Chr-7-G in UGT1A9, and 0.28 μM for Chr-4′-G in UGT1A1). The intrinsic clearance (CL_int_; *V*_max_/K_m_) of Dio-7-G, Dio-3′-G, Chr-7-G, and Chr-4′-G selectively in UGT1A6, UGT1A9, UGT1A9, and UGT1A1 were higher than that in HLMs.

**Table 2:** Kinetic parameters of diosmetin and chrysoeriol glucuronidation by human expressed UGT enzymes.

		K_m_ (μM)	*V*_max_	CL_int_
			nmol/mg/min	mL/mg/min
diosmetin	Dio-7-G (UGT1A6)	3.02±0.59	0.92±0.06	0.30
	Dio-3′-G (UGT1A9)	0.45±0.05	4.27±0.13	9.52
chrysoeriol	Chr-7-G (UGT1A9)	0.26±0.04	0.12±0.01	0.48
	Chr-4′-G (UGT1A1)	0.28±0.05	0.63±0.06	2.26

### Effects of Diosmetin and Chrysoeriol Concentrations on Glucuronide Excretion

**Fig 6:**
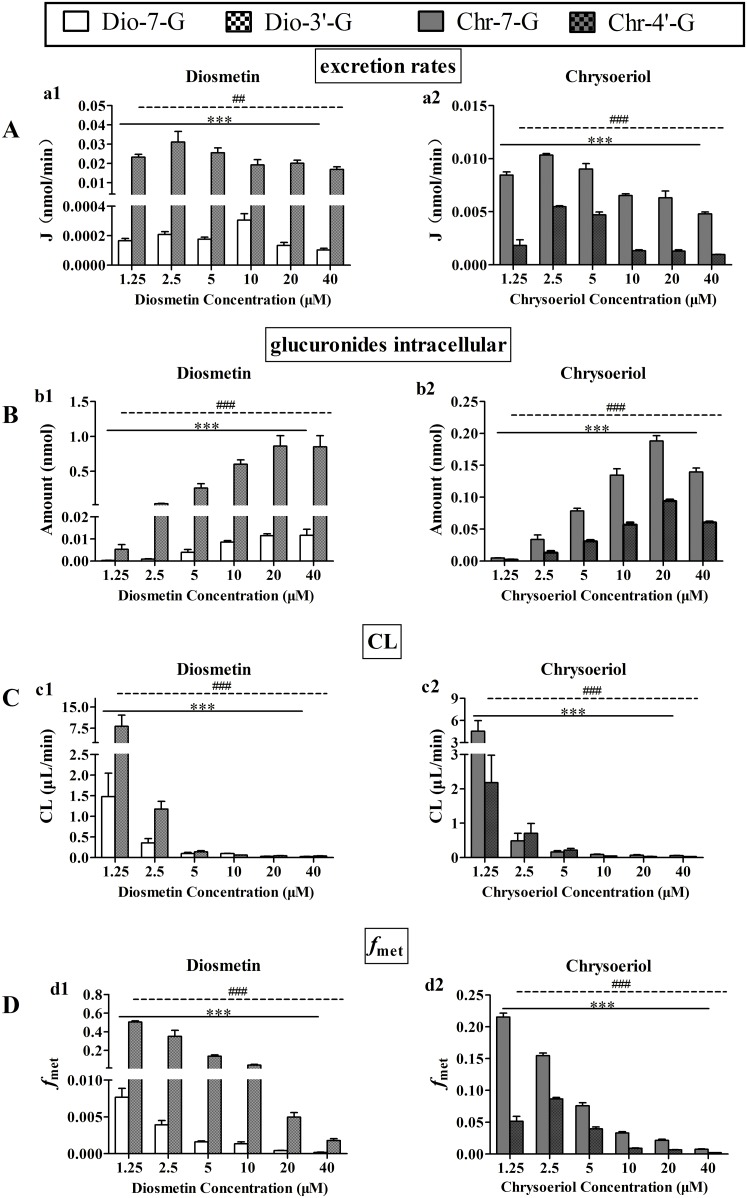
Effect of substrate concentrations on excretion rates (a), intracellular amounts (b), CL (c), and *f*_met_ (d) of Dio-7-G, Dio-3′-G, Chr-7-G, and Chr-4′-G. Three samples (500 μL) were obtained at 15, 30, and 60 min and replaced with fresh loading solution (500 μL) that contains diosmetin or chrysoeriol. The excretion rates of glucuronides were calculated from the slope of the amount-versus-time curves. The intracellular amounts of the glucuronides were determined at the end of the excretion experiments after the cells were washed twice with ice-cold HBSS. Each column corresponds to the average of three determinations with error bars representing the S.D. The “*” (for Dio-7-G or Chr-7-G) or “^#^” (for Dio-3′-G or Chr-4′-G) symbol means a statistically significant difference between-group, at p < 0.05; “**” or “^##^” means p < 0.01; “***” or “^###^” means p < 0.001.

Six concentrations of diosmetin and chrysoeriol were chosen to explore the effects of diosmetin and chrysoeriol concentrations on the excretion, intracellular amount, CL, and *f*_met_ in Hela and HeLa—UGT1A9 cells (Fig 6). Because it did not find the glucuronided metabolites of diosmetin or chrysoeriol in HeLa cells, we mainly determined the excretion, intracellular amount, CL, and *f*_met_ in HeLa—UGT1A9 cells. (Fig 6). The excretion rates of Dio-7-G reached a peak at 10 μM and Dio-3′-G reached a peak at 2.5 μM (Fig 6a1). The excretion rates of Chr-7-G and Chr-4′-G reached a peak at 2.5 μM, (Fig 6a2). Intracellular glucuronide levels dose-dependently increased with diosmetin or chrysoeriol loading concentrations (Fig 6b1 and 6b2). CL (Fig 6c1 and 6c2) dose-dependently decreased with increasing diosmetin or chrysoeriol concentration. And *f*_met_ (Fig 6d1 and 6d2) dose-dependently decreased with increasing diosmetin or chrysoeriol concentration except Chr-4′-G. The *f*_met_ of Chr-4′-G reached a peak at 2.5 μM chrysoeriol.

### Effect of Ko143 on Glucuronides Excretion

**Fig 7:**
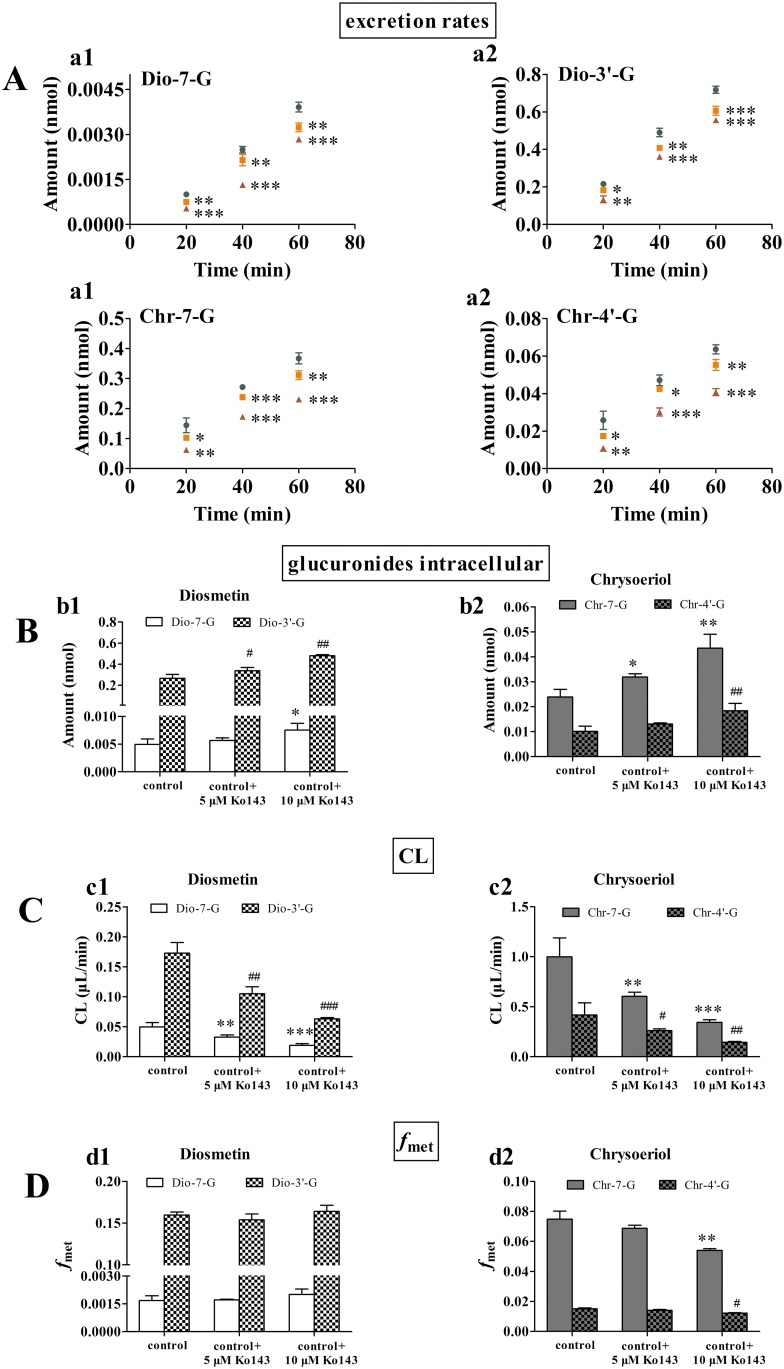
Effect of BCRP-specific inhibitor Ko143 on the excretion rate of glucuronides (Dio-7-G, Dio-3′-G, Chr-7-G, and Chr-4′-G) (a), total intracellular glucuronide amounts (Dio-7-G, Dio-3′-G, Chr-7-G, and Chr-4′-G) (b), CL (c), and *f*_met_ (d). Engineered HeLa cells stably overexpressing UGT1A9 grown on six-well plates (1 × 10^5^ cells/well) were treated with 2.5 μM of diosmetin or chrysoeriol in the absence or presence of Ko143 at 5 or 10 μM. Three samples (500 μL) were obtained at 20, 40, and 60 min and replaced with fresh loading solution (500 μL) containing 2.5 μM diosmetin or chrysoeriol. Each data point (panel a) and each column (panel b-d) corresponds to the average of three determinations with error bars representing the S.D. The “*” (for Dio-7-G or Chr-7-G) or “^#^” (for Dio-3′-G or Chr-4′-G) symbol means a statistically significant difference between-group at p < 0.05; “**” or “^##^” means p < 0.01; “***” or “^###^” means p < 0.001.

To estimate the effect of BCRP on the excretion of glucuronides in HeLa—UGT1A9 cells, the Ko143 was adopted (Fig 7). Due to at the excretion rate of diosmetin glucuronides or chrysoeriol glucuronides reached a peak at 2.5 μM substrate concentration, the concentration of substrate were set at 2.5 μM. Co-incubation of 5 μM or 10 μM Ko143 with diosmetin or chrysoeriol led to the excretion rates of diosmetin or chrysoeriol glucuronides decreased and their intracellular level significantly increased. Ko143 (5 μM) inhibited Dio-7-G and Dio-3′-G excretion by 19% and 16%, respectively; and Ko143 (10 μM) decreased the excretion by 40% and 29%, respectively (Fig 7a1 and 7a2). Moreover, 5 μM Ko143 inhibited Chr-7-G and Chr-4′-G excretion by 14% and 19%, respectively; and 10 μM Ko143 decreased both excretions by 43% (Fig 7a3 and 7a4). The effects of Ko143 on the total intracellular amount of glucuronides are displayed in Fig 7B. At 2.5 μM diosmetin, the total amount of intracellular Dio-7-G and Dio-3′-G was increased by 1.1- and 1.3-fold, respectively, in the presence of 5 μM Ko143 and by 1.5- and 1.8-fold, respectively, in the presence of 10 μM Ko143 compared with the control (Fig 7b1). At 2.5 μM chrysoeriol, the total amount of intracellular Chr-7-G and Chr-4′-G both increased by 1.3-fold in the presence of 5 μM Ko143 and by 1.7- and 1.8-fold, respectively, in the presence of 10 μM Ko143 compared with the control (Fig 7b2). Ko143 inhibited the CL of these four glucuronides (33% to 65%) (Fig 7C). The results of *f*_met_ (Fig 7D) indicated that the effect of Ko143 on diosmetin or chrysoeriol glucuronidation were small or insignificant. All the results indicated that the BCRP played a crucial role in excretion diosmetin glucuronides and chrysoeriol glucuronides.

### The effects of diosmetin and chrysoeriol on cell viability in A549 and HepG2 cells with untreated and treated Ko143

**Fig 8:**
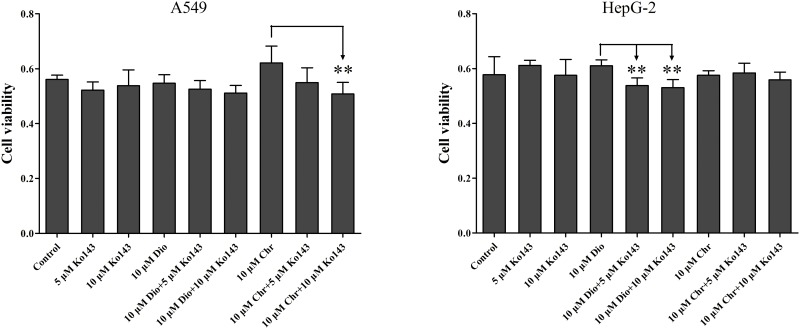
The effects of diosmetin and chrysoeriol on cell viability in A549 and HepG2 cells with untreated and treated Ko143. 5 μM Ko143, 10 μM Ko143, 10 μM diosmetin, 10 μM diosmetin with 5 μM Ko143 or 10 μM Ko143, 10μM chrysoeriol, 10μM chrysoeriol with 5 μM Ko143 or 10 μM Ko143 were used as substrates to determine the effects of diosmetin and chrysoeriol on cell viability in A549 and HepG2 cells with untreated and treated Ko143. Each column corresponds to the average of three determinations with error bars representing the S.D. The “*” symbol means a statistically significant difference compared with control at p < 0.05; “**” means p < 0.01; “***” means p < 0.001).

We found that in A549 cells, 10 μM chrysoeriol with 5 μM or 10 μM Ko143 respectively decreased the A549 cell viability by 12% and 18% compared with the group that substrate was 10μM chrysoeriol, but the cytotoxicity of diosmetin had little change when treated Ko143. And in HepG2 cells, 10 μM diosmetin with 5 μM or 10 μM Ko143 respectively decreased the A549 cell viability by 12% and 13% compared with the group that substrate was 10 μM diosmetin, but the cytotoxicity of chrysoeriol had little change when treated Ko143 (Fig 8).

## Discussion

Luteolin is widely found in our diet, such as carrots, peppers, celery, olive oil and peppermint [17]. It was reported that luteolin could be metabolized to diosmetin and chrysoeriol, which were the methylation metabolites. And the diosmetin and chrysoeriol could be metabolized in rats by Ugts [16]. Therefore, it was speculated that glucuronides of diosmetin and chrysoeriol would be formed in humans. The objective of this study was to the metabolism characteristic of diosmetin and chrysoeriol. The exposure of the glucuronides of diosmetin and chrysoeriol could influence the pharmacological action of luteolin. Hence, the metabolic elucidation of diosmetin and chrysoeriol is important to understand the pharmacokinetic characteristics and biological effects of luteolin *in vivo*. Diosmetin and chrysoeriol, which also distributed in many plant products, are pharmacologically valuable in many diseases, such as osteoporosis [4]. This study for the first time investigated the regioselectivity of the glucuronidation of diosmetin and chrysoeriol by kinetic profiling and identified the major enzymes responsible for the glucuronidation of the two compounds. In addition, this study also showed the effect of the BCRP—UGT1A9 interplay on diosmetin and chrysoeriol glucuronidation *in vivo*.

The metabolic activities of the HLMs toward diosmetin and chrysoeriol were determined by kinetic profiling (Fig 2). These activities included the metabolic rates at various substrate concentrations. The CL_int_ value, independent of substrate concentration, measures the catalytic efficiency of functional enzymes. The determination of regioselectivity was based on the CL_int_ derived from kinetic profiling over a wide range of substrate concentrations. The elucidation of regioselectivity by kinetic determination was superior to that by rate determination at high substrate concentrations [31]. In addition, the use of CL_int_ as indicator of UGT activity was more advantageous than the use of reaction rates because CL_int_ is more relevant in predicting hepatic clearance *in vivo* compared with other kinetic parameters [32,33]. In HLMs, the CL_int_ value of diosmetin through 3′-OH was much higher than that of the 7-OH, and the CL_int_ value of chrysoeriol through 4′-OH was about 32 times than that of the 7-OH pathway. For another, the K_m_ values of diosmetin and chrysoeriol glucuronides were lower than a series of 7-OH, 3’-OH or 4’-OH flavonoids, such as acacetin glucuronide (K_m_ was 14.465 μM)[34] and genistein glucuronides (K_m_ was 15.1 μM)[35] (Table 1).

On the other hand, human expressed UGTs and chemical inhibition assays were used to determine the major UGTs involved in the glucuronidation metabolism of diosmetin and chrysoeriol. Among the 12 expressed UGTs, only the enzymes from the UGT1A family except UGT1A4 showed glucuronidation activity on diosmetin and chrysoeriol, and the UGT2B family offered no contribution to their UGT metabolism. According to the expressed UGT assay, the main UGT isoforms for Dio-7-G, Dio-3′-G, Chr-7-G, and Chr-4′-G formation were UGT1A6, UGT1A9, UGT1A9 and UGT1A10, respectively (Fig 3). Because the expression of individual UGTs at the mRNA level in the liver are different (UGT1A1 (7%), UGT1A6 (5%), UGT1A9 (6%))[36], and the UGT isoform showing the highest activity could not be the main contributor in HLMs. Therefore, the inhibition assay was further performed to determine the role of the UGT isoform in the metabolism of diosmetin and chrysoeriol. In addition, it was reported that UGT1A8 and 1A10, which were involved in the glucuronidation of diosmetin and chrysoeriol, were not present in the liver [37]. And the objective of this research was to study the hepatic clearance of diosmetin and chrysoeriol. Thus, the effect of UGT1A8 and 1A10 was not further consideration. Therefore, UGT1A1, which was the second main isoform for Chr-4′-G formation, and UGT1A6, UGT1A9 were used to the further inhibition assay, and these three isoforms are highly expressed in liver [37]. Bilirubin (UGT1A1 inhibitor), phenylbutazone (UGT1A6 inhibitor) and carvacrol (UGT1A9 inhibitor) were used in this study. All the concentrations of diosmetin and chrysoeriol adopted in this study were based on the K_m_ value of glucuronides. The results showed that bilirubin, phenylbutazone, and carvacrol inhibited the formation of the glucuronides in UGT enzymes and HLMs in a dose-dependent manner (Fig 4). Thus, the results suggested that UGT1A6, UGT1A9, UGT1A9, and UGT1A1 were the main expressed UGTs responsible for the formation of Dio-7-G, Dio-3′-G, Chr-7-G, and Chr-4′-G, respectively. The inhibition kinetics of these three inhibitors have been previously described to be substrate dependent, but the mechanism of inhibition of drug-metabolizing enzymes remains unclear [29,38]. Thus, the mechanism of inhibition of these inhibitors requires additional studies, and *in vitro* data on the inhibition should be interpreted with caution. To further elucidate the characteristics of the glucuronidation metabolism of diosmetin and chrysoeriol, their rates of glucuronidation metabolism by UGT1A1, UGT1A6, and UGT1A9 were determined at different substrate concentrations (Fig 5). The results of comparable kinetic profiles of the expressed UGTs further explained the conclusion in chemical inhibition study (Table 2).

In this study, UGT1A9 was the main expressed UGTs responsible for the formation of Dio-3′-G and Chr-7-G, and Dio-7-G and Chr-4′-G also can be formed by UGT1A9. Therefore, the HeLa—UGT1A9 cells, which were used to study how the interplay between UGT1A9 and BCRP affects drug disposition [21], were utilized to further elaborate diosmetin and chrysoeriol metabolism in terms of glucuronide production. This work delineated the interplay between UGT1A9 and BCRP in diosmetin and chrysoeriol metabolism and disposition at the kinetic level. The excretion rates of Dio-7-G in HeLa—UGT1A9 peaked at 10 μM, and those of Dio-3′-G, Chr-7-G, and Chr-4′-G rapidly peaked at 2.5 μM (Fig 6). The glucuronide formation did not show substrate inhibition kinetics; hence, we speculated that the decrease in the excretion rates of the glucuronides at high diosmetin or chrysoeriol concentration was most likely caused by BCRP inhibition by diosmetin or chrysoeriol. Indeed, several flavonoids (e.g., ayanin and retusin) have been reported to inhibit BCRP encoded by the ABCG2 gene with potencies only slightly lower than that of Ko143. Structural features found to contribute positively to BCRP inhibition included a hydroxyl group in position 5 and a double bond between position 2 and 3 [39], which were also found in the structures of diosmetin and chrysoeriol. Therefore, BCRP inhibition by diosmetin or chrysoeriol was believed to account for the decreased excretion rate of glucuronides in the HeLa—UGT1A9 cells observed at high diosmetin or chrysoeriol concentrations.

The role of BCRP in the excretion was studied using Ko143, a specific BCRP inhibitor that does not affect UGT activities as a general rule. Ko143 (5 μM or 10 μM) significantly inhibited the BCRP-mediated excretion of the glucuronides of diosmetin and chrysoeriol. Ko143 also significantly increased the accumulation of intracellular glucuronides at 2.5 μM (Fig 7). Importantly, Ko143 inhibited glucuronide CL and *f*_met_ at 2.5 μM diosmetin or chrysoeriol, suggesting that BCRP-mediated excretion could be the predominant pathway for diosmetin and chrysoeriol disposition.

According to the results of the effect of Ko143 on glucuronides excretion, we found that the total intracellular amount of glucuronides was increases after treated Ko143. But the effects of increased intracellular glucuronides in cells were not clear. Therefore, we used these A549 and HepG2 tumor cell lines to demonstrate the effects of diosmetin and chrysoeriol on cell viability with untreated and treated Ko143 inhibitor (Fig 8). Both A549 and HepG2 cells express BCRP [40,41]. A549 cells express UGT1A1, UGT1A3 and UGT2B7 [42], and HepG2 cells expresse UGT1A1, UGT1A6 and UGT1A9 [43]. According to the present results, we found that the increased total intracellular glucuronides could increase the cytotoxicity, and diosmetin and chrysoeriol had different effects on different cancer cells, possibly due to different isoform of UGTs in the different cancer cells.

In conclusion, this study first demonstrated that the diosmetin and chrysoeriol could be metabolited into Dio-7-G, Dio-3′-G, Chr-7-G and Chr-4′-G by human in vitro. All the findings suggested that the oral bioavailability of luteolin, diosmetin and chrysoeriol would be greatly limited by first-pass glucuronidation in the liver. In addition, this work established that BCRP-mediated excretion was the predominant pathway for diosmetin and chrysoeriol disposition, which could help increase the understanding and prediction of BCRP-mediated glucuronide CL *in vivo*. Our studies contribute to improving the understanding on the pharmacokinetic characteristics and biological effects of luteolin *in vivo*.
